# Alteration in Levels of Specific miRNAs and Their Potential Protein Targets between Human Pancreatic Cancer Samples, Adjacent Normal Tissue, and Xenografts Derived from These Tumors

**DOI:** 10.3390/life13030608

**Published:** 2023-02-22

**Authors:** Fiona O’Neill, Taylor-Jade Allen-Coyle, Sandra Roche, Justine Meiller, Neil T. Conlon, Niall Swan, Robert M. Straubinger, Justin Geoghegan, Ninfa L. Straubinger, Kevin Conlon, Ray McDermott, Finbarr O’Sullivan, Michael Henry, Paula Meleady, Gerard McVey, Robert O’Connor, Michael Moriarty, Martin Clynes

**Affiliations:** 1 National Institute for Cellular Biotechnology, Dublin City University, D09 NR58 Dublin, Ireland; 2 SSPC, The Science Foundation Ireland Research Centre for Pharmaceuticals, V94 T9PX Limerick, Ireland; 3 St. Vincent’s University Hospital, D04 T6F4 Dublin, Ireland; 4 Department of Pharmaceutical Sciences, University at Buffalo, SUNY, Buffalo, NY 14214, USA; 5 School of Biotechnology, Dublin City University, D09 K2OV Dublin, Ireland; 6 St. Luke’s Hospital, Rathgar, D06 HH36 Dublin, Ireland

**Keywords:** pancreatic cancer, miRNA, PDX, tumour

## Abstract

Herein, we describe the global comparison of miRNAs in human pancreatic cancer tumors, adjacent normal tissue, and matched patient-derived xenograft models using microarray screening. RNA was extracted from seven tumor, five adjacent normal, and eight FI PDX tumor samples and analyzed by Affymetrix GeneChip miRNA 4.0 array. A transcriptome analysis console (TAC) was used to generate comparative lists of up- and downregulated miRNAs for the comparisons, tumor vs. normal and F1 PDX vs. tumor. Particular attention was paid to miRNAs that were changed in the same direction in both comparisons. We identified the involvement in pancreatic tumor tissue of several miRNAs, including miR4534, miR3154, and miR4742, not previously highlighted as being involved in this type of cancer. Investigation in the parallel mRNA and protein lists from the same samples allowed the elimination of proteins where altered expression correlated with corresponding mRNA levels and was thus less likely to be miRNA regulated. Using the remaining differential expression protein lists for proteins predicted to be targeted for differentially expressed miRNA on our list, we were able to tentatively ascribe specific protein changes to individual miRNA. Particularly interesting target proteins for miRs 615-3p, 2467-3p, 4742-5p, 509-5p, and 605-3p were identified. Prominent among the protein targets are enzymes involved in aldehyde metabolism and membrane transport and trafficking. These results may help to uncover vulnerabilities that could enable novel approaches to treating pancreatic cancer.

## 1. Introduction

Survival rates are very poor in pancreatic cancer, owing in part to the early disease being frequently asymptomatic. Diagnosis frequently occurs at an advanced stage when the tumor has invaded locally and often metastasized. While the majority of pancreatic adenocarcinomas (PDAC) are K-ras mutants [1], and there is as yet no clinically demonstrated way of selectively targeting it without also inhibiting wild-type Ras, which is essential for the survival of normal cells [2]. However, recent promising results with the covalent KRAS-G12C inhibitors sotorasib and adagrasib in the treatment of non-small cell lung cancer patients that harbor the mutation [3]. Many other mutations in proteins of signaling pathways have been identified in a proportion of pancreatic cancers, but to date, targeted therapies of the type that are so effective in, for example, molecularly defined subsets of breast cancer have not been defined in pancreatic cancer. A complex microenvironment with molecular crosstalk, for example, between stellate cells and cancer cells, complicates attempts to understand and treat pancreatic cancer [4]. There has been no fundamental advance in the treatment of pancreatic cancer in the past 20 years, although better medical management of side effects and improvements in surgery, pain control, and improved management of palliative quality of life indicators are all very important. However, a significant improvement in outcomes is still lacking [5]. This somewhat gloomy background underlines the need for a better understanding of the biology of pancreatic cancer in the hope of discovering new vulnerabilities that can be exploited in new treatments [6].

MicroRNAs are short, non-coding RNAs, typically of 20–24 nucleotides in length, that can be responsible for the regulation of biological pathways in cells. Since their discovery in the nematode C. elegans in 1993, these RNAs have received considerable attention due to their roles in diverse biological functions, including the regulation of cell development, differentiation, and survival. Approximately one-third of all human genes are thought to be regulated by miRNAs, with approximately 2500 miRNAs identified to date [7]. Dysregulation of miRNA profiles has been found to be associated with the development of various human cancers. In pancreatic cancer specifically, there has been a significant increase in interest in the expression of miRNAs and their possible roles as diagnostic or prognostic markers [8,9,10,11,12,13,14]. Some of these studies have highlighted particular miRNAs as being of particular interest, including miR21, miR217, and miR222. Furthermore, there has been an interest in the targeting of these miRNAs with antimirs as potential targets, although this has so far been met with limited success [15,16,17]. The stability of miRNAs in bodily fluids such as plasma or serum and tissue samples is a major advantage for their use as potential biomarkers, but lack of specificity may be a limitation [18].

In the work detailed in this paper, we have applied a multilevel approach looking at datasets from proteomic [19], mRNA microarray [20] or and miRNA microarray analysis from the same tumor-adjacent normal tissue (5 samples), tumor samples (7 samples) and F1 patient-derived xenograft (F1) tumor (8 samples) material cohorts. Applying this multilevel approach has allowed us to overcome one drawback often associated with miRNA studies. We have been able to identify effects on predicted targets of miRNAs using both mRNA and proteomic expression and have not had to rely on computational algorithms.

## 2. Materials and Methods

### 2.1. Acquisition and Generation of Patient-Derived Xenograft Models

Pancreatic tumor sample acquisition and generation of PDX models were described by our group [20]. Briefly, pancreatic tumor tissue (T) and adjacent normal tissue (N) were obtained from patients undergoing pancreatic ductal adenocarcinoma (PDAC) surgical resection at St. Vincent’s University Hospital Dublin. After initial macroscopic pathological confirmation, the material remaining after diagnostic sampling was cold transferred to DCU. The collection of patient material was approved by St Vincent’s University Hospital and Dublin City University (DCU) Research Ethics Committee. All animal work received ethical approval from the DCU Research Ethics Committee (DCUREC/2012/202) and was licensed by the Irish Department of Health (license number B100-4501). Severe combined immunodeficiency (SCID), CB17/lcr-Prkdc^scid^/lcrCrl mice (Charles River, U.K.) were implanted subcutaneously with fresh patient tumor material. Mice were monitored for tumor development for up to 1 year post-implantation. Animal welfare monitoring criteria included tumor volume, tumor axis, body weight, and condition. The tumor volume and tumor axis limits were set as <2000 mm^3^ and <20 mm, respectively. Following the humane euthanasia of the mouse, the tumor was divided for cryopreservation, formalin-fixed paraffin embedding (FFPE), and snap frozen (F1). For snap freezing, a piece of tumor was minced with a scalpel, placed in a cryovial, and placed directly into liquid nitrogen (LN_2_), then stored at −80 °C.

### 2.2. Proteomics Analysis

Proteomic analysis of PDAC tumors and normal tissue was previously described by our group [19]. Briefly, tissue samples were enriched for membrane proteins using Mem-PER plus membrane protein extraction kit as per the manufacturer’s instructions (Thermo Fisher, Hemel Hempsted, UK). Two subcellular fractions were generated, cytosolic and solubilized membrane/membrane-associated proteins. Nano-liquid chromatography-tandem mass spectrometry (LC-MS/MS) was carried out on a Dionex Ultimate 3000 nano-RSLC (Thermo Scientific, Hemel Hempsted, UK) coupled in-line to an Orbitrap Fusion Tribrid mass spectrometer (Thermo Scientific). The LC-MS/MS parameters were previously [21] described using a 140 min separation gradient per sample. Protein identification was carried out using Proteome Discoverer Version 2.2 software (Thermo Scientific) with the SEQUEST HT algorithm. Raw MS files were searched against the UniProt/SwissProt human protein database (downloaded on 29 August 2018, containing 20,325 sequences). The following criteria were applied: (1) precursor mass tolerance set to 10 ppm; (2) fragment mass tolerance set to 0.6 Da; (3) oxidation of methionine set as a dynamic modification; (4) carbamidomethylation of cysteine set as a static modification; and (5) a maximum of 2 missed cleavage sites were allowed. False-discovery rates were applied to peptide-spectrum matches using Percolator, and only those with a false-discovery rate of less than 1% were allowed. In the case of F1 samples, identified proteins could be of human or murine origin. From scrutiny of the peptides used to identify the proteins, we can divide the detected proteins into 3 categories: (i) human not murine, (ii) murine not human, (iii) could be murine or human. To simplify the analysis, we have confined our list to the first category—protein lists presented here are human not murine.

### 2.3. mRNA and miRNA Microarray Analysis

Sample preparation and mRNA microarray analysis was as previously described [20]. Briefly snap frozen samples of F1 tumors, original patient tumor material, and adjacent normal tissue were prepared for RNA extraction by grinding samples under LN_2_ using a mortar and pestle. The tissue sample was placed in a metal mortar with a small volume of LN_2_ and ground quickly. When powdered, the powder was transferred to a clean microcentrifuge tube. This was performed before the LN2 completely evaporated and while the powder was still frozen. Once homogenized, RNA samples were prepared using Trizol (Sigma, Merck KGaA, Darmstadt, Germany), according to the manufacturer’s guidelines. Samples were quantified using a Nanodrop (Thermo Scientific), and quality was determined using Agilent Bioanalyser (Agilent, Santa Clara, Ca, USA. Samples with RIN numbers greater than 8 were accepted as most suitable for microarray analysis.

Briefly, the preparation of cRNA, hybridization, and scanning of microarrays was performed according to the manufacturer’s protocol (Affymetrix, Thermo-Fischer, Santa Clara, CA, USA). A total of 300 ng of total RNA extracted from homogenized patient samples isolated using Trizol was converted into double-stranded cDNA by reverse transcription. Biotin-labeled cRNA was generated by converting the cDNA sample using the Genechip WT plus reagent kit (Affymetrix). Labeled cRNA was hybridized to the Affymetrix GeneChip^®^ Human Gene 2.0 ST Array while rotating at 60 rpm for 16 h at 45 °C. After hybridization, the microarray was washed using the Affymetrix Fluidics Station according to the manufacturer’s protocol. The chips were scanned in an Affymetrix 3000 7G scanner.

miRNA arrays were performed according to the manufacturer’s protocol (Affymetrix). A total of 1000 ng of total RNA was extracted from homogenized patient samples and isolated using Trizol. Following this, samples underwent Poly(A) tailing followed by flashtag biotin HSR ligation. Labeled samples were hybridized to the Affymetrix GeneChip^®^ miRNA 4.0 Array while rotating at 60 rpm for 16–18 h at 48 °C. After hybridization, the microarray was washed using the Affymetrix Fluidics Station according to the manufacturer’s protocol. The chips were scanned in an Affymetrix 3000 7G scanner.

### 2.4. Bioinformatic Analysis

Differential gene analysis expression was carried out using Applied Biosystems Transcriptome Analysis Console (TAC) software 4.0.2. The software was integrated with the established LIMMA package, which allowed for improved inference at both the gene and gene set levels in small experiments. Resulting gene signal values are expressed as biweight robust averages shown on the log2 scale. Fold change values are calculated from the log2 signal values. Resulting gene lists were filtered for ±1.5 fold changes, a *p*-value < 0.05 and an overall FDR F-Test: <0.005. The miRNA 4.0 chip comprises of all mature miRNA sequences in the miRBase release 20, including miRNA transcripts from humans, mice, and rats. As they are suitable for multi-species analysis, an additional filter of transcript ID containing only hsa was also included.

The miRNA and mRNA cel files are deposited on the GEO repository, submission GSE141873 (mRNA) and GSE207345 (miRNA).

miRwalk was used for predicted protein identification [22]. Filters applied to the results were a binding probability value of 0.95, a binding site position of 3-UTR, and a validated interaction with an miRTarbase entry.

## 3. Results

Bioinformatics analysis of the miRNA data from the tumor (T), adjacent normal (N), and patient-derived xenograft tumors (F1) identified 100 differentially expressed (DE) miRNA (87 upregulated and 13 downregulated in the tumor sample) in the tumor versus normal tissue samples (Supplementary Table S1). In the F1 vs. tumor comparison, 214 DE miRNA were identified (67 upregulated and 147 downregulated in the F1 samples (Supplementary Table S2). Identifications were based on a *p*-value < 0.05 and a fold change of ±1.5. A fold change of ±1.5 was selected as previous profiling studies have shown that even subtle changes in miRNA expression can have an impact on cell biology [23]. In comparisons where a very large fold change is recorded, it generally flags that the expression level is very low in one of the conditions rather than being a strictly accurate assessment of the ratio of the number of molecules present in each condition. These particular arrays (miRNA 4.0 array) have transcripts relating to an extensive range of species as well as humans, including mice and rats. There are a total of 4603 miRNA transcripts represented on the arrays that are determined to be expressed in humans. Due to the size and homogeneity of the miRNA sequences, it is not possible to say with certainty in the case of the F1 miRNA whether any particular miRNA is of human or mouse origin.

There are many ways in which the data could be presented and in which the three profile data strands could be analyzed. In this paper, we focus on (1) the changes of greatest magnitude up and down in the two comparisons (Table 1 and Table 2), (2) miRNAs where expression changed in the same direction in both comparisons (Table 3), and (3) changes in expression of individual proteins, which could be explained by our miRNA data (Table 4, Table 5 and Table 6).

### 3.1. Examination of miRNA Expression Changes in Pancreatic Cancer Tumor Samples and Adjacent Normal Tissue

**Figure 1:**
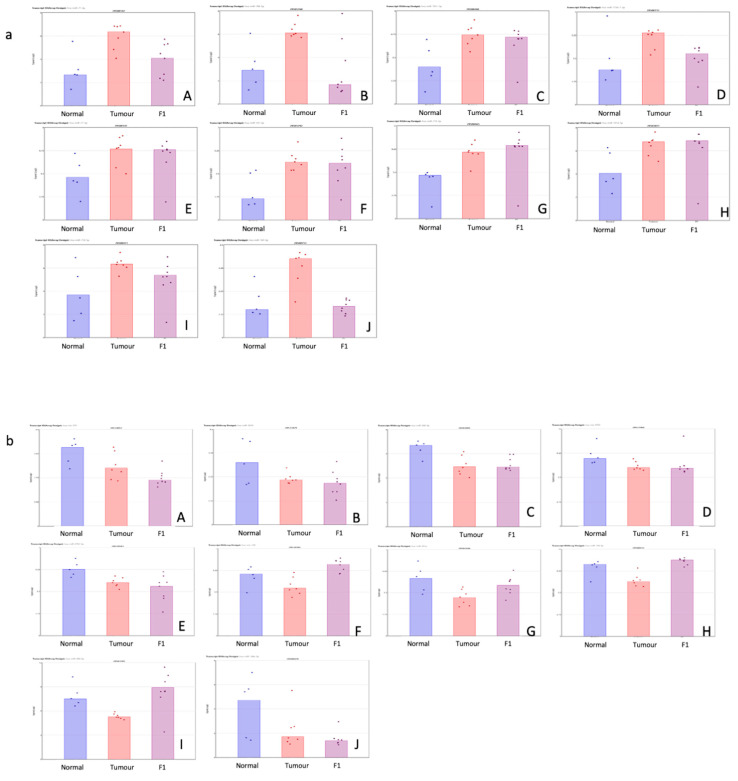
(**a**,**b**) Scatterplot representation of data from Table 1 (TvN comparison) to provide a visual representation of the interpatient variation of the expression of each miRNA in each of the 3 tissue cohorts. Column 1 represents adjacent normal tissue, column 2 represents the patient tumor material, and column 3 represents the F1 tissue. (**a**) Log2 expression scatterplots in normal, tumour and F1 conditions of miRNAs shown to be upregulated in TvN comparison (Table 1). (**A**) miR21-3p, (**B**) miR708-3P, (**C**) miR181c-5p, (**D**) miR125b-1-5p, (**E**) miR21-5p, (**F**) miR331-5p, (**G**)miR210-3p, (**H**) miR181d-5p, (**I**) miR214-5p and (**J**) miR143-5p. Each individual sample is represented by a dot in each condition and indicates the log2 expression of that miRNA. (**b**) Log2 expression scatterplots in normal, tumour and F1 conditions of miRNAs shown to be downregulated in TvN comparison (Table 1). (**A**) mir375, (**B**) miR3618, (**C**) miR509-5p, (**D**) mir6722, (**E**) miR4742-5p, (**F**) mir139, (**G**) miR451a, (**H**) miR139-5p, (**I**) miR486-5p and (**J**) miR148a-5p. Each individual sample is represented by a dot in each condition and indicates the log2 expression of that miRNA.

Table 1 shows the top 10 up- and downregulated miRNAs in the comparison of the patient tumor samples and adjacent normal tissue. Figure 1 shows a visual representation of each of the miRNA expressions in all three conditions within this study.

### 3.2. Examination of miRNA Expression Changes in Pancreatic Cancer Tumor Samples and Patient-Derived Xenograft Models

**Figure 2:**
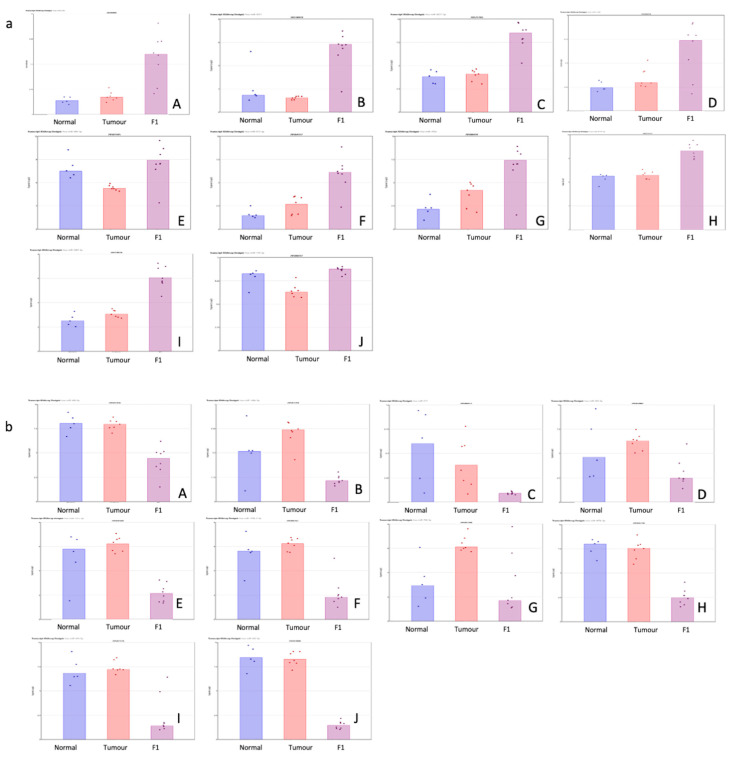
(**a**,**b**) Scatterplot representation of data from Table 1 (F1vT comparison) to provide a visual representation of the interpatient variation of the expression of each miRNA in each of the 3 tissue cohorts. Column 1 represents adjacent normal tissue, column 2 represents the patient tumor material, and column 3 represents the F1 tissue. (**a**) Log2 expression scatterplots in normal, tumour and F1 conditions of miRNAs shown to be upregulated in F1vT comparison (Table 2). (**A**) miR206, (**B**) miR4521, (**C**) miR6872-5P, (**D**) miR1290, (**E**) miR486-5p, (**F**) miR6153p, (**G**)miR203A, (**H**) miR6778-5p, (**I**) miR2467-3p and (**J**) miR139-5p. Each individual sample is represented by a dot in each condition and indicates the log2 expression of that miRNA. (**b**) Log2 expression scatterplots in normal, tumour and F1 conditions of miRNAs shown to be downregulated in F1vT comparison (Table 2). (**A**) miR409-3p, (**B**) miR146b-3p, (**C**) miR217, (**D**) miR503-5p, (**E**) miR1271-5p, (**F**) miR125b-2-3p, (**G**)miR708-5p, (**H**) miR487-3p, (**I**) miR424-5p and (**J**) miR432-5p. Each individual sample is represented by a dot in each condition and indicates the log2 expression of that miRNA.

Table 2 shows the top 10 up- and downregulated miRNAs in the comparison of the patient tumor samples and adjacent normal tissue. Figure 2 shows a visual representation of each of the miRNA expressions in all three conditions within this study.

### 3.3. Examination of miRNA Expression Changes in Pancreatic Cancer, Adjacent Normal Tissue, and Patient-Derived Xenograft Models of Pancreatic Cancer

**Figure 3:**
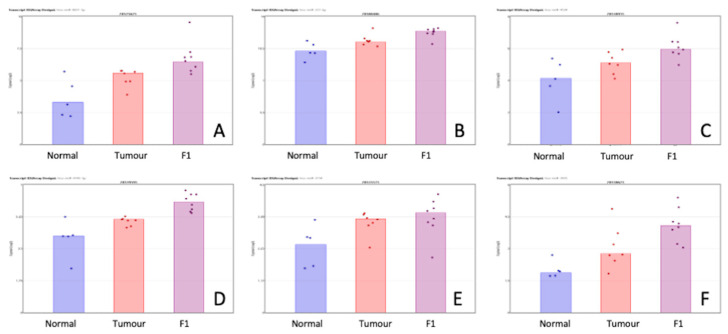
Scatterplot representation of data from Table 3 (miRNA shown to be differentially expressed in both the TvN and F1vT comparison) to provide a visual representation of the interpatient variation of the expression of each miRNA in each of the 3 tissue cohorts. Column 1 represents adjacent normal tissue, column 2 represents the patient tumor material, and column 3 represents the F1 tissue. Log2 expression scatterplots in normal tumour and F1 conditions of miRNAs shown to be upregulated in both the TvN and the F1vT comparison (Table 3). (**A**) miR6831-5p, (**B**) miR222-3p, (**C**) miR4534, (**D**) miR4743-5p, (**E**) miR3154, (**F**) miR3935. Each individual sample is represented by a dot in each condition and indicates the log2 expression of that miRNA.

Table 3 shows the priority miRNA that was shown to be differentially expressed in both comparisons in the same direction. Figure 3 show a visual representation of each of the miRNA expression in all three conditions within this study. While the comparisons TvN and F1vT are clearly interesting in their own right, we have also searched for changes that happen in both comparisons but in the same direction. These may be of special interest because the F1 tumor may be due to cell selection following transplantation, have a higher proportion of tumor cells than the original tumor sample (a mixture of normal and tumor cells), and our proteomics analysis identifies only human and not murine proteins. Of course, other phenomena are also involved in these transitions; for example, major changes in the cellular composition of the stroma.

### 3.4. Investigation of Predicted miRNA Target Proteins in Proteomic and Transcription Datasets

Since miRNAs target multiple mRNAs and proteins, we hypothesized that these DE expression lists might lead us to pathways as well as individual proteins of functional, diagnostic, or therapeutic significance. We are particularly well placed to investigate this network in detail in this study as we have performed global mRNA [20] and proteomic [19,24] profiling on the same clinical and F1 samples. Protein changes that do not have a corresponding same direction mRNA change could be strong candidates for regulation by miRNAs. Histological examples, as well as clinical descriptions of the samples, were previously described previously [20].

Table 4 (TvN), Table 5 (F1vT), and Table 6 (DE in the same direction in both) list predicted protein targets of specific miRNAs, which, as well as being predicted, were also found in the proteomic dataset to be different from the expressed in the appropriate direction (i.e., inverse to the direction of difference of expression of the miRNA). Note that only proteins for which the mass spectrometry data indicated definitively human, and not murine, origin are included in our analysis to avoid confusion in the interpretation of the F1 proteomic data.

The predicted protein list was generated from miRWalk, which was filtered to only include validated interactions with an entry on miRTarbase [22].

Of the 39 miRNAs investigated from the priority list, 7 miRNAs are enriched in the list by targeting multiple proteins. miRs 2467, 4742, 509, 615-3p, 4534, 222, and 206 are all shown to target two or more proteins across the two comparisons.

## 4. Discussion

The core concept of this study was to use the differential expression of miRNAs as a tool to further unravel the biology of human pancreatic cancer in the hope of uncovering new targets or vulnerabilities that might be exploited therapeutically; and/or finding new biomarkers or combinations of biomarkers that might contribute to better or earlier diagnosis [25]. We believe that the study of miRNAs may be particularly fruitful since each miRNA targets multiple mRNAs, regulating the cellular levels of the corresponding proteins

The data presented here can be viewed in a number of different ways, simple lists of miRNAs DE between tumor and normal and between F1 and tumors (and in some cases both), and because of our previously published information on DE mRNAs and proteins, it is also possible to tentatively identify predicted protein targets of specific DE miRNAs.

Previous analyses of these proteomic [19,24] and mRNA [20] or datasets on these clinical and F1 samples have looked at each dataset individually. mRNA analysis [20] showed an enrichment of genes associated with proliferation, cell cycle, and mitotic processes. Label-free LC-MS-based proteomic analysis carried out on this sample set identified a number of membrane proteins in both comparisons (TvN, F1vT) that had the potential to be utilized either as biomarkers or ADC drug conjugate targets [24].

The study of miRNAs in the development, progression, and prognosis of PDAC has intensified in the last decade. Interestingly there are few studies including transcriptomic and proteomic analysis as well as miRNA analysis on the same sample set. This limitation has meant that protein and gene targets of interesting miRNAs have predominantly been identified using predictive software and have not been validated in matched samples [8,25,26,27,28,29]. Mattie et al. [30] performed miRNA analysis on matched tumor vs. PDX samples; however, this study was complicated by a high degree of homology with mouse miRNA sequences. Hanoun et al. [31] initially published a study looking at the silencing of miR148a as a potential therapeutic target in PDAC. However, following further studies in which they looked at the role this miRNA had in the modulation of protein expression, they discovered that miR148a was responsible for only minimal protein regulation and were unable to identify protein targets in cell lines [32]. This indicates the importance of looking at parallel analysis where possible, as it may help to reduce the number of false leads emerging from in silico studies [33] miR148a was shown to be significantly downregulated in our patient tumor when compared with the normal-adjacent samples fold change of −8.15, it was, however, unchanged in the F1vT comparison. The miRWalk prediction software identified nine targets for this miRNA; however, when compared with the protein list, none of these were inversely expressed.

The investigation of miRNAs, with mRNA and proteomic investigations in parallel from the same samples, as we propose, may lead to the identification of proteins that could be druggable targets. Furthermore, RNA therapeutics are now a reality, and miRNA manipulation has the potential to become a useful addition to the therapeutic armamentarium against pancreatic cancer [15,34].

### 4.1. Tumor vs. Adjacent Normal Tissue Comparison

Examination of the dysregulation of miRNA in our dataset provided a short list of interesting miRNA; the top 10 up and down differentially expressed miRNAs in the (a) TvN comparison (Table 1), (b) F1vT comparisons (Table 2), and (c) miRNAs that are differentially expressed in the same direction in both comparisons (Table 3). Of the 39 miRNAs within this priority list, 7 of them predicted an effect on multiple proteins, which were found to be inversely expressed in the corresponding protein dataset (Table 4, Table 5 and Table 6). Several studies in gastric cancer [35], triple-negative breast cancer [36], and lung cancer [37] have indicated roles for miR509. This miRNA, however, has not been implicated in the literature on pancreatic cancer. Our data indicated that miR509 was shown to be downregulated in the patient tumors when compared to the normal adjacent, with a fold change of −1.85 (Supplementary Table S1). It was predicted to target a number of proteins in our list, including COTL1, LRPAP1, and SL4A2 (Table 4). Predicted targets of the remaining miRNAs were shown to be differentially expressed in the TvN comparisons, although none of their predicted targets were found to be inversely expressed in the proteomic dataset, but alterations in the expression of miR708 [38], miR210 [39,40], and miR214 [41,42] have all been studied in pancreatic cancer.

In the TvN comparison, it would be expected that we would see the alteration in the expression of proteins and miRNAs associated with tumorigenesis and proliferation in the pathways associated with this transition. miR21 has been extensively studied in PDAC, with high expression levels being linked with shorter overall survival [43,44,45], while miR331-5p has not been reported in any cancer-linked study. miR21-5p and miR331-5p were shown to target Dimethylarginine Dimethylaminohydrolase 1 (DDAH1). Reduced levels of DDAH1 have been shown to mediate cell invasion and metastasis in gastric cancer through the WNT signaling pathway. Additionally, downregulation was linked with poor prognosis [46]. These miRNAs were both shown to be upregulated in the TvN comparison, and the expression of DDAH1 was shown to be downregulated in the membrane protein fraction of the same comparison. Our data suggest that both miR181c and miR181d target phosphatidylethanolamine binding protein 1 (PEPB1) with a reduction in expression in the TvN in both the membrane and cytoplasmic fraction. Loss of expression of PEBP1 (also known as raf kinase inhibitor protein (RKIP), an endogenous inhibitor of the MAPK pathway) has been shown to be linked with PDAC metastasis and poor overall survival [47,48].

miR210-3p was predicted to target endoplasmic reticulum protein 27 (ERP27), and the protein was shown to be inversely expressed in the membrane fraction (Table 4). Transcriptomic analysis of the cancer genome atlas has identified ERP27 as a pivotal gene in the pathogenesis and progression of PDAC [49]. Increased glutathione S-transferase M3 (GSTM3) expression has been linked with improved overall survival in PDAC [50]. This protein was inversely regulated by miR143-5p, which was found to be upregulated in the tumor samples when compared with the normal-adjacent samples. Literature searches showed no previous studies linked with cancer and the expression of miR4742. Our proteomic data shows that miR4742 targets a number of proteins within the dataset, including superoxide dismutase 2 (SOD2), cysteine and glycine-rich protein 1 (CSRP1), and protein S100-A16 (S100A16). SOD2 is involved in oxidative phosphorylation and the regulation of ROS (reactive oxygen species), and upregulation in its expression has been linked with acquired resistance to ROS-inducing anticancer drugs and potential irradiation in pancreatic cancer cell lines [51]. While little is known about CSRP1 in pancreatic cancer, S100A16 has been extensively studied in PDAC. Roles in metastasis [52], epithelial–mesenchymal transition (EMT) [53], and increased expression linked with poor prognosis have all been reported in the literature. In our protein data, we do see an increased expression in the membrane fraction in both the TvN and F1vT comparison of S100A16.

### 4.2. F1 Xenograft vs. Tumor Comparison

In the case of the F1vT comparison, our previous studies on mRNA and protein expression changes on the samples that were used here have highlighted the importance of proteins and mRNA that drive the important process of tumor engraftment in tumorigenesis. As described by Coleman et al. [24], the selection pressure that is exerted on the tumor cells during the engraftment process may result in the altered expression of proteins that drive tumor growth. The upregulation of miR125a in the PDAC samples agrees with what is already shown in the literature [54], and it has been shown that its expression can contribute to cell survival [55]. One of its predicted targets is SGT1 homolog (SUGT1), a highly conserved protein generally found in the nucleus, involved in the kinetochore function and essential for the G1/S and G2M transition, which was shown to be increased in expression in the cytosolic fraction of the tumors in comparison to the tumor sample., The expression of this protein has been potentially linked with a poor prognosis in colorectal cancer [56].

Keklikoglou et al., 2015, found that the expression of miR206 was downregulated in pancreatic tumors vs. non-malignant normal tissue [57]. In our study, miR206 was not differentially expressed in the TvN comparison; however, it was significantly upregulated in the F1vT comparison with a fold change of 26.01. Wu et al. showed that the expression of miR-206 inhibited hepatocellular carcinoma cell migration and invasion while promoting apoptosis by targeting Peptidylprolyl Isomerase B (PPIB) [58]. Another study evaluated the expression of 41 genes as a function of in vitro radioresistance in the NCI-60 cancer cell line panel and found that PPIB had the strongest direct correlation. They also showed that siRNA downregulation of PPIB leads to radio-sensitization of the cancer cells [59]. PPIB expression was shown to be downregulated in both comparisons (TvN and F1vT) in our proteomic data.

Levels of miR2467 were shown to be significantly increased in the F1 samples in comparison to the original patient tumor. This miRNA was predicted to target a large number of proteins, and when examined in our protein dataset, a large number of proteins showed inverse expression. There are limited studies in the literature on this miRNA, with one indicating a downregulation of the miRNA in colorectal tumor samples compared to tumor-adjacent normal tissue [60]. We observed a decrease in the expression of Aflatoxin B1 aldehyde reductase member 2 (AKR7A2) in both of our comparisons, not reported in the proteomic analysis by Cui et al. [61]. Furthermore, this protein was found to be targeted by two miRNAs in our analysis (miR2467 and miR1290). The latter has been suggested as a biomarker in PDAC [62]. Wei et al. [63] and Tavaano et al. [64] both examined the expression of miR1290, with and without CA19-9 in serum and plasma, respectively, and noted that while the expression was found to be higher in PDAC patients, it alone was insufficient to select patients at risk of developing PDAC. Both studies indicated that combining the expression of miR1290 with Ca19-9 was more effective. Whole miRNome and proteome profiling revealed miR1290 as a novel hypoxia-associated microRNA, which was highly abundant in hypoxic extracellular vesicles (EVs) and also exhibited a signature consisting of six proteins including AKR7A2, which was significantly associated with a poor prognosis for melanoma patients [65].

miR615 overexpression has been shown to inhibit cell proliferation, migration, and invasion in vitro [66]. miR615 was increased in the F1 samples compared to the original patient tumors, and a number of predicted target proteins were found to be inversely expressed in the matching transition in proteomic data. These include several enzymes involved in the reduction and oxidation of aldehydes (AKR7A2, aldehyde dehydrogenase 1 family member B1 (ALDH1B1). Aldehyde dehydrogenase (ALDH2)) and aldehyde dehydrogenases are often highly expressed in cancer stem cells. ALDH1B1 may have a role in cell proliferation [67]. It has also been suggested as a potential biomarker of colon cancer [68]. It was another predicted target of miR615 found in our proteomic dataset to be downregulated in the F1vT comparison as well as in the cytosolic fraction of the TvN comparison. Calnexin (CANX), a member of the calnexin family of molecular chaperones, is a calcium-binding, endoplasmic reticulum (ER)-associated protein that interacts transiently with newly synthesized N-linked glycoproteins, facilitating protein folding and assembly. It may also play a central role in the quality control of protein folding by retaining incorrectly folded protein subunits within the ER for degradation. This protein has the potential as a biomarker in breast [69] and lung patients [70]. Several of the other miR615-3p targets identified in our analysis showed consistent changes across both comparisons and are involved in processing membrane protein targets to ER (SECG1A1, SPC53, TM95F2).

The miR-378 family has been associated with numerous cancers, including: osteosarcoma [71], cervical [72], and colon [73]. MiR-378e has not yet been extensively studied for an oncogenic/oncosuppressive role. Wang et al. [73] have reported an association between miR-378e and colorectal cancer, as the survival rate of patients in the high-expression group was significantly higher than that in the low-expression group. In our dataset, it was shown to be differentially upregulated in the F1 samples vs. the patient samples. miR409 expression, which was downregulated in the F1 tumors vs. patient tumor samples, has been linked with PDAC cell proliferation, invasion, and migration [74] proteins. Predicted to be targeted by miR409, Catenin Delta 1 (CTNND1) along with Catenin alpha 1 (CTNNA1) and Catenin Beta 1 (CTNNB1) expression was shown to indicate a poor prognosis for pancreatic cancer patients [75]. In the F1 samples, this protein was shown to be differentially upregulated versus the patient samples (Table 5).

### 4.3. miRNA Altered in Both Comparisons

Across the two transitions, from normal to the tumor and then tumor to F1 models, there was a small cohort of miRNAs that were shown to be DE, namely miR4534, miR4743, miR222, miR3154, and miR6831. miR-6831-5p had been previously identified as being differentially expressed in PDAC vs. normal control serum samples, as shown by Aita et al. [76]. KEGG pathway analysis showed enrichment for the pancreatic cancer term. Dysregulation of miR6831-5p has been associated with gastric [77] and colorectal cancer [78]. Additionally, PLD3, which we found to be downregulated in both the cytoplasmic and membrane protein fraction of the F1 tumor samples compared to the patient material, has been shown to be a potential marker of senescence [79]. Limited studies have been completed on miR4534; however, one study indicated the potential roles of miR4534 in prostate cancer through the regulation of PTEN [80]. miR3154 has been investigated in leukemia, with its expression shown to be higher at initial diagnosis compared to complete remission [81]. miR222 has been widely studied in pancreatic cancer. Elevated levels of these miRNAs have been associated with poor survival [82,83,84,85].

Of course, a caveat with any research of this kind using tissue extracts is the fact that the samples consist of a heterogenous mixture of cell types and connective tissue, so we cannot, for example, be certain that an alteration observed in the tumor samples reflects an alteration in expression in the tumor cells (for definitive attribution some form of in situ hybridization would be needed for miRNAs and mRNAs, and immunohistochemistry in the case of proteins).

## 5. Conclusions

This profiling study complements the data currently available on the expression of miRNA in pancreatic cancer. The availability of the mRNA microarrays and LC-MS-MS proteomic data has allowed for the tentative validation of what would typically have only been predicted targets. By combining the three datasets, we have been able to identify connections between the expression of proteins and their miRNA regulators that have not been identified in pancreatic cancer previously. Analysis of the F1 samples may also allow for the identification of miRNA, which has roles in key networks associated with tumor engraftment and proliferation. The combined analysis has provided a shortlist of protein and miRNAs that warrant further investigation; this list has a mixture of established miRNAs associated with PDAC progression, in particular, miR21, miR2467, miR615 but also a number of miRs that have either limited or no studies in pancreatic cancer such as miR4534, miR3154, and miR4742. Nevertheless, some of these may have potential as biomarkers or for the development of therapeutic targets with further validation. Further analysis of these three parallel data strands that we have placed in the public domain by others using new bioinformatic tools could discover additional connections and networks that we could not uncover by our analysis. The lists we make available here will then be available to other groups for histochemical investigation and for validation in a variety of cancer-relevant in vitro assays for functional assignment.

## Figures and Tables

**Table 1:** Priority miRNA from miRNA array analysis with corresponding fold changes and *p*-values for TvN comparison.

	TvN	
Transcript ID (Array Design)	Fold Change	*p*-Values
hsa-miR-21-3p	12.89	1.20 × 10^−3^
hsa-miR-708-5p	9.15	6.70 × 10^−3^
hsa-miR-181c-5p	8.52	3.00 × 10^−3^
hsa-miR-125b-1-3p	6.95	3.18 × 10^−2^
hsa-miR-21-5p	6.89	1.58 × 10^−2^
hsa-miR-331-5p	6.81	3.60 × 10^−3^
hsa-miR-210-3p	6.73	6.60 × 10^−3^
hsa-miR-181d-5p	6.51	2.28 × 10^−2^
hsa-miR-214-5p	6.3	1.02 × 10^−2^
hsa-miR-143-5p	5.55	2.00 × 10^−4^
hsa-mir-375	−1.67	3.16 × 10^−2^
hsa-miR-3618	−1.76	4.70 × 10^−2^
hsa-miR-509-5p	−1.85	1.30 × 10^−3^
hsa-mir-6722	−2	4.62 × 10^−2^
hsa-miR-4742-5p	−2.06	2.15 × 10^−2^
hsa-mir-139	−2.2	4.92 × 10^−2^
hsa-miR-451a	−2.93	3.50 × 10^−3^
hsa-miR-139-5p	−4.51	1.20 × 10^−3^
hsa-miR-486-5p	−4.65	1.33 × 10^−2^
hsa-miR-148a-5p	−8.15	4.08 × 10^−2^

**Table 2:** Priority miRNA from miRNA array analysis with corresponding fold changes and *p*-values for F1vT comparison.

	F1vT	
Transcript ID (Array Design)	Fold Change	*p*-Values
hsa-miR-206	26.01	8.59 × 10^−5^
hsa-miR-4521	24.27	4.48 × 10^−6^
hsa-miR-6872-5p	21.19	1.35 × 10^−7^
hsa-miR-1290	15.4	6.80 × 10^−3^
hsa-miR-486-5p	12.48	1.40 × 10^−3^
hsa-miR-615-3p	10.81	5.59 × 10^−5^
hsa-miR-203a	9.43	2.30 × 10^−3^
hsa-miR-6778-5p	8.56	5.42 × 10^−8^
hsa-miR-2467-3p	7.91	2.01 × 10^−8^
hsa-miR-139-5p	6.7	2.78 × 10^−6^
hsa-miR-409-3p	−11.45	1.47 × 10^−6^
hsa-miR-146b-3p	−12.7	6.39 × 10^−6^
hsa-miR-217	−13.39	1.98 × 10^−2^
hsa-miR-503-5p	−14.24	2.30 × 10^−3^
hsa-miR-1271-5p	−17.01	1.88 × 10^−6^
hsa-miR-125b-2-3p	−21.33	6.58 × 10^−7^
hsa-miR-708-5p	−21.66	2.00 × 10^−3^
hsa-miR-487b-3p	−34.33	1.31 × 10^−9^
hsa-miR-424-3p	−56.66	2.27 × 10^−6^
hsa-miR-432-5p	−113.44	2.10 × 10^13^

**Table 3:** Priority miRNA from miRNA array analysis with corresponding fold changes and *p*-values for shown to be differentially expressed in both comparisons in the same direction.

	TvN		F1vT	
Transcript ID (Array Design)	Fold Change	*p*-Val	Fold Change	*p*-Values
hsa-miR-6831-5p	4.82	2.16 × 10^−2^	1.81	1.32 × 10^−2^
hsa-miR-222-3p	1.99	7.10 × 10^−3^	2.27	2.41 × 10^−2^
hsa-miR-4534	1.92	4.76 × 10^−2^	1.83	3.49 × 10^−2^
hsa-miR-4743-5p	1.87	1.12 × 10^−2^	1.94	2.20 × 10^−3^
hsa-miR-3154	1.86	3.12 × 10^−2^	5.14	1.60 × 10^−5^
hsa-miR-3935	1.85	2.99 × 10^−2^	2.5	1.59 × 10^−2^

**Table 4:** Priority miRNA fold changes and *p*-values and corresponding predicted protein target expression identified in LC-Ms/MS dataset with *p*-value for the TvN comparison.

	TvN		F1vT			TvN (Membrane)		TvN (Cytosolic)		F1vT (Membrane)		F1vT (Cytocolic)	
Transcript ID (Array Design)	FC	*p*-Val	FC	*p*-Values	Gene Symbol	FC	*p*-Values	FC	*p*-Values	FC	*p*-Values	FC	*p*-Values
hsa-miR-143-5p	5.55	2.00 × 10^−4^			GSTM3	−2.04	6.48 × 10^−3^					−15.16	6.46 × 10^−3^
hsa-miR-181c-5p	8.52	3.00 × 10^−3^			PEBP1	−3.20	1.19 × 10^−2^	−2.90	1.33 × 10^−3^	−2.10	1.04 × 10^−2^	−3.50	3.77 × 10^−5^
hsa-miR-181d-5p	6.51	2.28 × 10^−2^			PEBP1	−3.20	1.19 × 10^−2^	−2.90	1.33 × 10^−3^	−2.10	1.04 × 10^−2^	−3.50	3.77 × 10^−5^
hsa-miR-210-3p	6.73	6.60 × 10^−3^			ERP27	−21.19	9.29 × 10^−4^						
hsa-miR-214-5p	6.3	1.02 × 10^−2^			TBL2			−13.95	4.01 × 10^−3^				
hsa-miR-21-5p	6.89	1.58 × 10^−2^			DDAH1	−2.13	1.92 × 10^−3^					4.16	3.84 × 10^−4^
hsa-miR-331-5p	6.81	3.60 × 10^−3^			DDAH1	−2.13	1.92 × 10^−3^					4.16	3.84 × 10^−4^
hsa-miR-4742-5p	−2.06	2.15 × 10^−2^			CSRP1	3.11	1.39 × 10^−3^	2.34	1.31 × 10^−2^				
hsa-miR-4742-5p	−2.06	2.15 × 10^−2^			S100A16	4.20	1.12 × 10^−2^			3.57	8.51 × 10^−4^		
hsa-miR-4742-5p	−2.06	2.15 × 10^−2^			SOD2	2.29	5.88 × 10^−3^			−4.84	5.06 × 10^−6^	−22.02	2.29 × 10^−9^
hsa-miR-509-5p	−1.85	1.30 × 10^−3^			COTL1	2.23	3.83 × 10^−2^					8.77	1.61 × 10^−4^
hsa-miR-509-5p	−1.85	1.30 × 10^−3^			LRPAP1	2.21	3.10 × 10^−3^			−2.37	1.53 × 10^−3^		
hsa-miR-509-5p	−1.85	1.30 × 10^−3^			SLC4A2	6.03	3.11 × 10^−3^			−1.98	5.14 × 10^−3^		

List of predicted target proteins for priority miRNAs in Table 1 list, which also showed inverse DE with the miRNA in the proteomics analysis.

**Table 5:** Priority miRNA fold changes and *p*-values and corresponding predicted protein target expression identified in LC-Ms/MS dataset with *p*-value for the F1vT comparison.

	TvN		F1vT			TvN (Membrane)		TvN (Cytosolic)		F1vT (Membrane)		F1vT (Cytocolic)	
Transcript ID (Array Design)	FC	*p*-Values	FC	*p*-Values	Gene Symbol	FC	*p*-Values	FC	*p*-Values	FC	*p*-Values	FC	*p*-Values
hsa-miR-125b-2-3p			−21.3	6.58 × 10^−7^	SUGT1							3.19	9.56 × 10^−4^
hsa-miR-206			26.01	8.59 × 10^−5^	PPIB			−3.55	5.45 × 10^−3^	−1.94	2.55 × 10^−2^	−3.30	1.64 × 10^−2^
hsa-miR-206			26.01	8.59 × 10^−5^	SLC25A22	−2.77	1.19 × 10^−2^						
hsa-miR-615-3p			10.81	5.59 × 10^−5^	DPP3	2.53	2.66 × 10^−3^			−1.91	1.20 × 10^−2^	2.25	3.86 × 10^−4^
hsa-miR-615-3p			10.81	5.59 × 10^−5^	GANAB	1.67	7.26 × 10^−3^			−2.04	2.12 × 10^−4^		
hsa-miR-708-5p			−21.7	2.00 × 10^−3^	RANBP2							1.93	8.16 × 10^−3^
hsa-miR-2467-3p			7.91	2.01 × 10^−8^	AKR7A2			−2.09	1.56 × 10^−3^	−1.81	1.01 × 10^−2^		
hsa-miR-2467-3p			7.91	2.01 × 10^−8^	ATP1B3	1.88	3.03 × 10^−2^			−2.26	1.59 × 10^−2^		
hsa-miR-2467-3p			7.91	2.01 × 10^−8^	CPM					−5.55	1.31 × 10^−2^		
hsa-miR-2467-3p			7.91	2.01 × 10^−8^	FKBP15					−4.64	5.12 × 10^−3^		
hsa-miR-2467-3p			7.91	2.01 × 10^−8^	RAB1A			−3.33	4.87 × 10^−3^				
hsa-miR-2467-3p			7.91	2.01 × 10^−8^	UGGT1					−1.80	1.91 × 10^−2^		
hsa-miR-378e			4.2	1.10 × 10^−3^	TXNL1					−2.54	2.30 × 10^−3^		
hsa-miR-6778-5p			8.56	5.42 × 10^−8^	ATP5F1A	−2.19	9.76 × 10^−3^	−2.70	1.53 × 10^−3^				

List of predicted target proteins for priority miRNAs in Table 2 list, which also showed inverse DE with the miRNA in the proteomics analysis.

**Table 6:** Priority miRNA fold changes and *p*-values and corresponding predicted protein target expression identified in LC-Ms/MS dataset with *p*-value for miRNA that were DE expressed in the same direction in both comparisons.

	TvN		F1vT			TvN (Membrane)		TvN (Cytosolic)		F1vT (Membrane)		F1vT (Cytocolic)	
Transcript ID (Array Design)	FC	*p*-Values	FC	*p*-Values	Gene Symbol	FC	*p*-Values	FC	*p*-Values	FC	*p*-Val	FC	*p*-Values
hsa-miR-4534	1.92	4.76 × 10^−2^	1.83	3.49 × 10^−2^	CALR					−2.18	1.52 × 10^−4^		
hsa-miR-4534	1.92	4.76 × 10^−2^	1.83	3.49 × 10^−2^	CAPZA1					−2.05	2.49 × 10^−3^		
hsa-miR-4743-5p	1.87	1.12 × 10^−2^	1.94	2.20 × 10^−3^	CYP20A1					−2.50	6.46 × 10^−3^		
hsa-miR-222-3p	1.99	7.10 × 10^−3^	2.27	2.41 × 10^−2^	GNAI3					−2.56	3.85 × 10^−4^		
hsa-miR-3154	1.86	3.12 × 10^−2^	5.14	1.60 × 10^−5^	HSP90B1	−6.63	4.34 × 10^−3^			−1.72	1.11 × 10^−2^	−2.71	
hsa-miR-4534	1.92	4.76 × 10^−2^	1.83	3.49 × 10^−2^	OLA1	−4.41	3.21 × 10^−4^						
hsa-miR-6831-5p	4.82	2.16 × 10^−2^	1.81	1.32 × 10^−2^	PLD3					−3.55	1.13 × 10^−3^	−6.41	
hsa-miR-222-3p	1.99	7.10 × 10^−3^	2.27	2.41 × 10^−2^	RECK					−12.65	9.53 × 10^−8^		
hsa-miR-222-3p	1.99	7.10 × 10^−3^	2.27	2.41 × 10^−2^	SOD2	2.29	5.88 × 10^−3^			−4.84	5.06 × 10^−6^	−22.02	
hsa-miR-222-3p	1.99	7.10 × 10^−3^	2.27	2.41 × 10^−2^	TOM1					−1.93	3.90 × 10^−2^		

List of predicted target proteins for priority miRNAs in Table 3 list, which also showed inverse DE with the miRNA in the proteomics analysis.

## Data Availability

The microarray data have been deposited in the NCBI’s Gene Expression Omnibus (GEO) under GEO series accession no GSE141873 (mRNA) and GSE207345 (miRNA). Proteomic data used in the analysis is available in Supplementary Files from cited publications [18,21]. Additional datasets used and/or analyzed during the current study may be requested from the corresponding author on reasonable request.

## Supplementary Materials

The following supporting information can be downloaded at: https://www.mdpi.com/article/10.3390/life13030608/s1, Table S1: List of differentially expressed (DE) miRNA in tumour vs adjancent normal tissue comparison, Table S2: List of differentially expressed (DE) miRNA in F1 vs. tumour comparison.
