# Supplementary material for: Alteration in Levels of Specific miRNAs and Their Potential Protein Targets between Human Pancreatic Cancer Samples, Adjacent Normal Tissue, and Xenografts Derived from These Tumors
Source: Life (Basel). 2023 Feb 22;13(3):608. doi: 10.3390/life13030608 (PMC10057657; doi:10.3390/life13030608)
Supplement: Supplementary file 1 [file life-13-00608-s001.zip › life-2120826-supplementary.pdf]

Supplementary *Table S1: List of differentially expressed (DE) miRNA in Tumour vs adjacent normal tissue comparison*

**Transcript ID(Array Design)**	**Tumour Avg (log2)**	**Normal Avg (log2)**	**Fold Change**	**P-val**
hsa-miR-21-3p	6.37	2.68	12.89	1.20E-03
hsa-miR-708-5p	6.11	2.92	9.15	6.70E-03
hsa-miR-181c-5p	6.69	3.6	8.52	3.00E-03
hsa-miR-125b-1-3p	5.43	2.64	6.95	3.18E-02
hsa-miR-21-5p	6.94	4.15	6.89	1.58E-02
hsa-miR-331-5p	4.39	1.62	6.81	3.60E-03
hsa-miR-210-3p	7.9	5.15	6.73	6.60E-03
hsa-miR-181d-5p	6.79	4.08	6.51	2.28E-02
hsa-miR-214-5p	6.35	3.69	6.3	1.02E-02
hsa-miR-143-5p	3.84	1.36	5.55	2.00E-04
hsa-miR-6831-5p	5.58	3.32	4.82	2.16E-02
hsa-miR-941	4.58	2.42	4.47	3.20E-02
hsa-miR-125a-3p	5.68	3.54	4.4	1.84E-02
hsa-miR-5195-3p	4.24	2.22	4.07	1.22E-02
hsa-miR-505-3p	4.71	2.71	4	1.88E-02
hsa-miR-874-3p	7.71	5.73	3.96	3.30E-02
hsa-miR-664b-5p	4.66	2.68	3.94	2.80E-03
hsa-let-7d-3p	4.36	2.42	3.84	2.41E-02
hsa-miR-34c-3p	3.74	1.92	3.54	1.08E-05
hsa-miR-766-3p	4.39	2.62	3.4	3.45E-02
hsa-miR-1231	6.16	4.51	3.14	2.08E-02
hsa-miR-4269	7.08	5.47	3.06	2.30E-02
hsa-miR-6851-5p	4.08	2.48	3.04	3.90E-03
hsa-miR-146b-3p	5.18	3.61	2.96	4.01E-02
hsa-mir-941-1	4.31	2.79	2.87	1.12E-02
hsa-mir-941-2	4.31	2.79	2.87	1.12E-02
hsa-mir-941-3	4.31	2.79	2.87	1.12E-02
hsa-mir-941-4	4.31	2.79	2.87	1.12E-02
hsa-miR-6890-5p	3.99	2.49	2.83	1.10E-03
hsa-miR-4725-3p	3.84	2.42	2.66	3.00E-03
hsa-miR-24-2-5p	3.38	2	2.6	4.70E-03
hsa-miR-4726-5p	3.39	2.05	2.52	5.30E-03
hsa-miR-6165	5.78	4.45	2.52	2.04E-02
hsa-miR-8071	3.64	2.31	2.51	1.53E-02
hsa-miR-1292-5p	4.28	2.98	2.47	1.46E-02
hsa-miR-6769a-5p	4.21	2.92	2.46	2.50E-03
hsa-miR-1296-5p	4.78	3.49	2.44	4.88E-02
hsa-mir-21	4.55	3.26	2.43	5.80E-03
hsa-miR-371a-5p	3.85	2.58	2.41	6.20E-03
hsa-miR-5010-5p	4.1	2.85	2.37	4.07E-02
hsa-miR-8060	4.05	2.82	2.34	5.40E-03
hsa-miR-492	2.82	1.66	2.24	1.65E-02

hsa-mir-3653	4.44	3.28	2.24	1.06E-02
hsa-miR-3187-3p	4.41	3.25	2.23	2.07E-02
hsa-miR-665	5.58	4.46	2.17	4.34E-02
hsa-miR-6508-5p	3.36	2.28	2.11	1.16E-02
hsa-miR-221-3p	11.19	10.12	2.1	4.20E-03
hsa-miR-214-3p	12.47	11.4	2.09	4.46E-02
hsa-miR-6757-5p	4.8	3.75	2.07	1.70E-02
hsa-miR-6785-5p	4.96	3.94	2.03	1.47E-02
hsa-miR-5088-5p	2.59	1.58	2.02	1.08E-02
hsa-miR-874-5p	3.77	2.77	2.01	1.17E-02
hsa-miR-6766-5p	2.51	1.51	1.99	5.70E-03
hsa-miR-222-3p	11.22	10.23	1.99	7.10E-03
hsa-miR-198	2.59	1.6	1.99	4.69E-02
hsa-miR-6845-5p	4.94	3.97	1.96	1.75E-02
hsa-mir-345	2.94	1.97	1.95	3.48E-02
hsa-miR-6124	5.94	4.98	1.94	2.90E-03
hsa-miR-4657	2.67	1.72	1.94	1.90E-03
hsa-miR-23a-3p	13.41	12.45	1.94	1.09E-02
hsa-miR-4534	5.09	4.15	1.92	4.76E-02
hsa-miR-542-5p	2.42	1.49	1.91	4.98E-02
hsa-mir-210	2.21	1.28	1.9	6.00E-04
hsa-miR-4743-5p	5.1	4.2	1.87	1.12E-02
hsa-miR-3154	3.3	2.4	1.86	3.12E-02
hsa-miR-3935	2.77	1.88	1.85	2.99E-02
hsa-miR-181a-5p	12.43	11.54	1.85	7.60E-03
hsa-miR-6830-5p	3.96	3.12	1.79	2.08E-02
hsa-miR-4800-3p	2.83	1.99	1.79	2.23E-02
hsa-let-7i-5p	12.16	11.32	1.79	2.82E-02
hsa-miR-760	2.46	1.66	1.74	5.40E-03
hsa-miR-7152-3p	4.34	3.55	1.72	3.37E-02
hsa-miR-7851-3p	2.61	1.82	1.72	1.29E-02
hsa-miR-6792-5p	2.84	2.06	1.72	3.38E-02
hsa-miR-145-5p	13.67	12.9	1.71	4.50E-03
hsa-miR-6862-5p	2.38	1.62	1.7	4.00E-03
hsa-miR-6080	2.39	1.71	1.6	1.88E-02
hsa-miR-6795-5p	4.33	3.66	1.59	3.49E-02
hsa-miR-4722-5p	2.75	2.08	1.59	4.46E-02
hsa-mir-663b	3.19	2.52	1.59	3.68E-02
hsa-miR-6895-5p	2.53	1.88	1.57	9.50E-03
hsa-miR-4778-5p	4.16	3.51	1.57	4.05E-02
hsa-miR-187-5p	3.27	2.64	1.55	1.16E-02
hsa-mir-887	1.85	1.24	1.52	1.79E-02
hsa-miR-4750-3p	2.12	1.52	1.52	1.99E-02
hsa-miR-181c-3p	1.97	1.37	1.51	1.50E-02
hsa-miR-4283	1.83	1.25	1.5	2.30E-03
hsa-miR-548x-3p	3.63	4.22	-1.51	2.95E-02

hsa-miR-4287	1.71	2.41	-1.63	1.87E-02
hsa-mir-548e	1.31	2.02	-1.64	1.10E-02
hsa-mir-375	2.11	2.85	-1.67	3.16E-02
hsa-miR-3618	2.11	2.92	-1.76	4.70E-02
hsa-miR-509-5p	2.47	3.35	-1.85	1.30E-03
hsa-mir-6722	6.65	7.64	-2	4.62E-02
hsa-miR-4742-5p	4.23	5.27	-2.06	2.15E-02
hsa-mir-139	3.83	4.97	-2.2	4.92E-02
hsa-miR-451a	3.12	4.67	-2.93	3.50E-03
hsa-miR-139-5p	6.92	9.1	-4.51	1.20E-03
hsa-miR-486-5p	5.28	7.5	-4.65	1.33E-02
hsa-miR-148a-5p	1.7	4.73	-8.15	4.08E-02

Supplementary *Table S2: List of differentially expressed (DE) miRNA in PDX F1 samples vs tumour samples comparison*

**Transcript ID(Array Design)**	**F1 Avg (log2)**	**Tumour Avg (log2)**	**Fold Change**	**P-val**
hsa-miR-206	6.58	1.88	26.01	8.59E-05
hsa-miR-4521	5.85	1.25	24.27	4.48E-06
hsa-miR-6872-5p	8.5	4.1	21.19	1.35E-07
hsa-miR-1290	6.62	2.67	15.4	6.80E-03
hsa-miR-486-5p	8.93	5.28	12.48	1.40E-03
hsa-miR-615-3p	6.11	2.68	10.81	5.59E-05
hsa-miR-203a	7.44	4.2	9.43	2.30E-03
hsa-miR-6778-5p	9.99	6.89	8.56	5.42E-08
hsa-miR-2467-3p	6.06	3.08	7.91	2.01E-08
hsa-miR-139-5p	9.67	6.92	6.7	2.78E-06
hsa-miR-29b-1-5p	6.94	4.39	5.82	2.64E-02
hsa-miR-6754-3p	4.23	1.7	5.77	2.00E-04
hsa-miR-4656	8.51	6.03	5.59	2.07E-06
hsa-miR-138-5p	4.83	2.36	5.55	1.60E-02
hsa-mir-3154	7.48	5.12	5.14	1.60E-05
hsa-miR-4298	10.15	7.8	5.09	1.59E-05
hsa-miR-4485	9.94	7.62	5.01	5.10E-03
hsa-miR-196a-5p	4.1	1.89	4.64	4.00E-03
hsa-miR-378d	7.16	5.01	4.44	4.30E-02
hsa-miR-378e	4.15	2.08	4.2	1.10E-03
hsa-miR-652-3p	10.28	8.26	4.06	1.35E-07
hsa-miR-378i	8.51	6.57	3.84	2.84E-02
hsa-miR-133a-3p	3.46	1.53	3.83	2.35E-02
hsa-mir-139	5.75	3.83	3.77	7.02E-05
hsa-miR-378g	6.35	4.45	3.72	3.91E-02

hsa-miR-378c	9.39	7.51	3.67	9.00E-04
hsa-miR-3911	6.56	4.88	3.21	1.40E-03
hsa-miR-211-5p	2.78	1.1	3.21	1.11E-02
hsa-miR-378a-3p	10.67	9.11	2.95	1.29E-05
hsa-mir-1973	3.51	2.02	2.8	6.00E-04
hsa-mir-6872	3.11	1.67	2.71	7.41E-05
hsa-miR-215-5p	2.94	1.52	2.67	1.21E-02
hsa-miR-34c-5p	3.75	2.36	2.62	1.74E-02
hsa-miR-3935	4.09	2.77	2.5	1.59E-02
hsa-miR-612	3.91	2.59	2.5	2.40E-02
hsa-miR-7847-3p	9.86	8.55	2.49	1.20E-03
hsa-miR-4669	6.98	5.69	2.45	6.50E-03
hsa-miR-7162-3p	6.65	5.37	2.44	8.60E-03
hsa-miR-4484	9.09	7.9	2.28	9.30E-03
hsa-miR-222-3p	12.4	11.22	2.27	2.41E-02
hsa-miR-133b	2.59	1.42	2.25	4.38E-02
hsa-miR-1910-3p	4.85	3.68	2.25	2.72E-02
hsa-miR-6723-5p	6.2	5.15	2.07	6.40E-03
hsa-miR-4448	2.84	1.84	2	4.62E-02
hsa-miR-451a	4.11	3.12	1.98	2.64E-02
hsa-miR-93-5p	11.09	10.13	1.95	2.70E-02
hsa-miR-4743-5p	6.06	5.1	1.94	2.20E-03
hsa-miR-34a-5p	9.72	8.79	1.9	3.65E-02
hsa-miR-4801	3.12	2.21	1.87	1.80E-03
hsa-miR-4534	5.96	5.09	1.83	3.49E-02
hsa-miR-6504-5p	2.04	1.18	1.81	1.03E-02
hsa-miR-6831-5p	6.44	5.58	1.81	1.32E-02
hsa-mir-31	2.63	1.8	1.77	1.88E-02
hsa-miR-4445-3p	3.12	2.31	1.75	1.44E-02
hsa-mir-3123	2.81	2.04	1.71	1.30E-03
hsa-miR-196b-5p	2.04	1.27	1.71	1.31E-02
hsa-mir-4485	3.96	3.21	1.69	3.90E-03
hsa-miR-140-3p	10.25	9.53	1.65	1.68E-02
hsa-miR-6880-5p	6.45	5.75	1.63	3.62E-02
hsa-miR-210-5p	2.89	2.19	1.62	3.70E-03
hsa-mir-3935	2.64	1.95	1.62	1.32E-02
hsa-miR-16-5p	11.58	10.89	1.61	4.66E-02
hsa-miR-15b-5p	11.24	10.58	1.58	8.60E-03
hsa-miR-298	2.14	1.49	1.57	4.61E-02
hsa-miR-6819-5p	7.65	7.02	1.55	3.15E-02
hsa-miR-4793-3p	1.94	1.31	1.55	2.05E-02
hsa-miR-718	2.47	1.88	1.5	4.30E-02
hsa-miR-320c	12.23	12.82	-1.5	1.17E-02
hsa-miR-6503-5p	1.1	1.69	-1.51	8.40E-03
hsa-mir-320a	7.82	8.42	-1.51	1.05E-02
hsa-miR-4792	1.34	1.94	-1.52	8.30E-03

hsa-miR-383-5p	1.36	1.96	-1.52	2.43E-02
hsa-miR-6511a-5p	4.14	4.74	-1.52	1.44E-02
hsa-miR-4787-5p	12.47	13.07	-1.52	1.46E-02
hsa-mir-4477a	1.79	2.41	-1.54	1.00E-02
hsa-miR-570-5p	1.44	2.06	-1.54	2.54E-02
hsa-miR-548ai	1.44	2.06	-1.54	2.54E-02
hsa-miR-132-5p	1.3	1.93	-1.54	1.91E-02
hsa-mir-4477a	1.77	2.4	-1.54	2.80E-03
hsa-mir-155	1.64	2.3	-1.58	2.01E-02
hsa-miR-628-5p	3.75	4.41	-1.58	1.80E-02
hsa-miR-329-3p	1.17	1.83	-1.58	2.06E-02
hsa-miR-3656	11.08	11.74	-1.59	2.44E-02
hsa-miR-4725-5p	1.38	2.06	-1.6	1.01E-02
hsa-miR-6784-5p	3.98	4.68	-1.62	3.15E-02
hsa-miR-199b-5p	1.24	1.95	-1.63	8.50E-03
hsa-mir-6836	4.48	5.2	-1.64	2.14E-02
hsa-miR-99b-5p	10.95	11.66	-1.64	1.93E-02
hsa-miR-6084	1.46	2.18	-1.65	1.46E-02
hsa-miR-638	11.1	11.83	-1.65	3.21E-02
hsa-mir-572	1.92	2.64	-1.65	1.70E-03
hsa-miR-6125	11.56	12.28	-1.65	7.00E-03
hsa-let-7f-1-3p	1.63	2.35	-1.65	3.50E-03
hsa-mir-487b	1.02	1.75	-1.67	3.00E-04
hsa-miR-6075	6.49	7.24	-1.68	1.76E-02
hsa-miR-320d	11.09	11.84	-1.68	1.55E-02
hsa-miR-1273h-5p	3.43	4.19	-1.7	3.86E-02
hsa-miR-4638-5p	1.89	2.67	-1.72	1.16E-02
hsa-mir-548ag-1	2.92	3.7	-1.72	2.71E-02
hsa-miR-543	1.79	2.6	-1.75	3.86E-02
hsa-miR-323a-3p	1.88	2.69	-1.76	6.20E-03
hsa-miR-3187-3p	3.6	4.41	-1.76	4.26E-02
hsa-mir-145	2.06	2.88	-1.77	1.12E-05
hsa-miR-665	4.76	5.58	-1.77	2.45E-02
hsa-miR-3122	2.04	2.87	-1.78	3.64E-02
hsa-miR-1227-5p	8.4	9.25	-1.8	3.69E-02
hsa-miR-125b-5p	12.74	13.6	-1.81	2.30E-03
hsa-miR-1273f	2.49	3.38	-1.85	6.90E-03
hsa-miR-7108-5p	9.97	10.86	-1.86	1.24E-02
hsa-miR-3185	7.39	8.29	-1.86	2.16E-02
hsa-miR-129-2-3p	1.76	2.66	-1.87	1.05E-02
hsa-miR-7851-3p	1.7	2.61	-1.88	9.80E-03
hsa-miR-6737-5p	3.24	4.18	-1.92	1.90E-02
hsa-miR-6790-5p	6.39	7.33	-1.92	3.02E-02
hsa-miR-130a-3p	7.05	7.99	-1.93	1.25E-02
hsa-miR-34c-3p	2.77	3.74	-1.97	9.00E-04
hsa-miR-6511b-3p	1.33	2.31	-1.97	2.22E-02

hsa-miR-132-3p	8.55	9.53	-1.98	1.65E-02
hsa-miR-381-3p	2.02	3.02	-2	4.64E-02
hsa-mir-487b	0.85	1.86	-2.01	2.00E-04
hsa-miR-1825	1.96	2.98	-2.03	2.37E-02
hsa-miR-887-3p	1.41	2.45	-2.05	4.60E-03
hsa-miR-4467	9.23	10.27	-2.06	3.14E-02
hsa-miR-6754-5p	2.31	3.35	-2.06	2.32E-02
hsa-miR-4417	5.61	6.68	-2.09	3.62E-02
hsa-miR-650	2.27	3.35	-2.12	5.60E-03
hsa-miR-214-3p	11.38	12.47	-2.13	3.01E-02
hsa-miR-4646-5p	4.28	5.39	-2.16	2.81E-02
hsa-miR-3917	2.04	3.16	-2.16	4.10E-02
hsa-miR-542-5p	1.31	2.42	-2.17	2.10E-03
hsa-miR-2861	10.2	11.34	-2.2	3.00E-03
hsa-miR-23c	3.75	4.9	-2.21	4.99E-02
hsa-miR-494-3p	7.91	9.05	-2.21	3.66E-02
hsa-miR-493-5p	1.19	2.34	-2.21	7.40E-03
hsa-miR-5572	4.37	5.52	-2.22	3.50E-02
hsa-miR-199a-3p	10.08	11.24	-2.25	1.03E-02
hsa-miR-199b-3p	10.08	11.24	-2.25	1.03E-02
hsa-miR-574-3p	9.87	11.05	-2.26	5.60E-03
hsa-miR-1304-3p	1.35	2.55	-2.3	1.00E-04
hsa-miR-874-3p	6.5	7.71	-2.32	3.32E-02
hsa-mir-424	2.83	4.06	-2.35	1.80E-03
hsa-mir-3687	2.85	4.09	-2.35	5.60E-03
hsa-miR-491-5p	3.52	4.76	-2.36	4.08E-02
hsa-miR-197-3p	7.44	8.7	-2.41	1.14E-02
hsa-miR-1291	2.06	3.33	-2.41	1.30E-02
hsa-miR-3615	2.58	3.9	-2.5	9.30E-03
hsa-miR-7641	10.2	11.53	-2.52	2.40E-02
hsa-miR-940	3.13	4.47	-2.53	1.10E-03
hsa-mir-125b-2	0.85	2.2	-2.55	2.00E-04
hsa-miR-3195	7.54	8.9	-2.56	2.87E-02
hsa-miR-375	10.11	11.49	-2.59	4.15E-02
hsa-miR-6511a-3p	2.13	3.54	-2.66	3.90E-03
hsa-miR-6508-5p	1.86	3.36	-2.82	1.00E-03
hsa-miR-874-5p	2.27	3.77	-2.84	7.00E-04
hsa-miR-193a-5p	7.86	9.38	-2.86	1.59E-02
hsa-miR-4730	5.49	7.01	-2.88	2.65E-02
hsa-miR-1270	1.55	3.09	-2.91	2.27E-02
hsa-miR-125b-1-3p	3.86	5.43	-2.98	2.26E-02
hsa-miR-150-5p	8.14	9.73	-3.01	2.70E-02
hsa-miR-487a-3p	1.35	2.99	-3.11	1.40E-03
hsa-miR-146b-5p	6.62	8.27	-3.14	5.00E-04
hsa-miR-6068	6.66	8.32	-3.16	2.00E-03
hsa-miR-4310	1.52	3.2	-3.21	7.00E-04

hsa-miR-504-5p	1.46	3.17	-3.27	3.53E-02
hsa-miR-409-5p	2.33	4.06	-3.32	4.14E-02
hsa-miR-99a-5p	8.58	10.31	-3.32	1.10E-03
hsa-miR-337-5p	1.41	3.15	-3.34	3.00E-04
hsa-miR-134-5p	5.37	7.11	-3.34	2.80E-03
hsa-miR-199a-5p	8.7	10.49	-3.45	4.00E-04
hsa-mir-3653	2.65	4.44	-3.46	8.00E-04
hsa-miR-4269	5.29	7.08	-3.46	5.00E-03
hsa-miR-6069	2	3.82	-3.51	2.40E-03
hsa-miR-766-3p	2.55	4.39	-3.58	1.60E-03
hsa-miR-1238-3p	1.49	3.37	-3.68	2.10E-03
hsa-miR-193b-3p	8.03	9.95	-3.78	1.37E-02
hsa-miR-145-5p	11.73	13.67	-3.83	1.90E-08
hsa-miR-143-3p	8.48	10.43	-3.87	1.07E-05
hsa-miR-505-3p	2.73	4.71	-3.95	5.80E-03
hsa-miR-664b-5p	2.65	4.66	-4.03	8.50E-05
hsa-miR-1281	5.22	7.26	-4.11	3.60E-03
hsa-miR-4324	2.66	4.74	-4.22	3.00E-03
hsa-miR-572	4.46	6.55	-4.24	1.60E-03
hsa-miR-3687	2.78	4.91	-4.38	6.70E-03
hsa-miR-193b-5p	4.2	6.37	-4.51	4.30E-02
hsa-miR-654-5p	1.45	3.69	-4.72	2.00E-04
hsa-miR-21-3p	4.1	6.37	-4.81	9.20E-03
hsa-miR-485-3p	3.43	5.71	-4.88	5.40E-03
hsa-miR-143-5p	1.52	3.84	-4.97	1.42E-05
hsa-miR-664b-3p	3.05	5.42	-5.16	4.11E-02
hsa-miR-100-5p	7.69	10.09	-5.27	2.00E-04
hsa-miR-5571-5p	1.98	4.39	-5.3	4.00E-04
hsa-miR-155-5p	8.04	10.47	-5.4	1.11E-02
hsa-miR-377-5p	1.26	3.72	-5.49	6.00E-04
hsa-miR-1247-5p	2.84	5.31	-5.55	5.10E-03
hsa-miR-493-3p	1.17	3.78	-6.1	3.85E-06
hsa-miR-371b-5p	3.77	6.39	-6.11	6.00E-04
hsa-miR-191-3p	2.16	4.79	-6.2	4.22E-05
hsa-miR-485-5p	3.27	5.98	-6.58	1.70E-03
hsa-miR-370-3p	2.78	5.55	-6.8	1.00E-04
hsa-miR-1296-5p	1.99	4.78	-6.92	4.00E-04
hsa-miR-654-3p	1.15	4.17	-8.14	4.27E-05
hsa-miR-216b-5p	3.07	6.32	-9.49	1.86E-02
hsa-miR-487a-5p	1.29	4.66	-10.3	4.86E-05
hsa-miR-1301-3p	3.11	6.6	-11.22	1.80E-03
hsa-miR-409-3p	4.45	7.96	-11.45	1.47E-06
hsa-miR-146b-3p	1.51	5.18	-12.7	6.39E-06
hsa-miR-217	1.19	4.94	-13.39	1.98E-02
hsa-miR-503-5p	2.47	6.31	-14.24	2.30E-03
hsa-miR-1271-5p	2.13	6.22	-17.01	1.88E-06

hsa-miR-125b-2-3p	1.83	6.25	-21.33	6.58E-07
hsa-miR-708-5p	1.68	6.11	-21.66	2.00E-03
hsa-miR-487b-3p	2.48	7.58	-34.33	1.31E-09
hsa-miR-424-3p	1.41	7.24	-56.66	2.27E-06
hsa-miR-432-5p	1.46	8.29	-113.44	2.10E-13
